# Parkinson's disease resting tremor severity classification using machine learning with resampling techniques

**DOI:** 10.3389/fnins.2022.955464

**Published:** 2022-10-28

**Authors:** Asma Channa, Oana Cramariuc, Madeha Memon, Nirvana Popescu, Nadia Mammone, Giuseppe Ruggeri

**Affiliations:** ^1^Department of Computer Science, University POLITEHNICA of Bucharest, Bucharest, Romania; ^2^Department of Information Engineering, Infrastructure and Sustainable Energy, Mediterranea University of Reggio Calabria, Reggio Calabria, Italy; ^3^IT Center for Science and Technology (CITST), Bucharest, Romania; ^4^Department of Computer Systems, Mehran University of Engineering and Technology, Jamshoro, Pakistan; ^5^Department of Civil, Energy, Environmental Engineering, Mediterranea University of Reggio Calabria, Reggio Calabria, Italy

**Keywords:** Parkinson's disease, resting tremor, imbalance data, resampling techniques, accelerometer data, machine learning, severity analysis

## Abstract

In resting tremor, the body part is in complete repose and often dampens or subsides entirely with action. The most frequent cause of resting tremors is known as idiopathic Parkinson's disease (PD). For examination, neurologists of patients with PD include tests such as finger-to-nose tests, walking back and forth in the corridor, and the pull test. This evaluation is focused on Unified Parkinson's disease rating scale (UPDRS), which is subjective as well as based on some daily life motor activities for a limited time frame. In this study, severity analysis is performed on an imbalanced dataset of patients with PD. This is the reason why the classification of various data containing imbalanced class distribution has endured a notable drawback of the performance achievable by various standard classification learning algorithms. In this work, we used resampling techniques including under-sampling, over-sampling, and a hybrid combination. Resampling techniques are incorporated with renowned classifiers, such as XGBoost, decision tree, and K-nearest neighbors. From the results, it is concluded that the Over-sampling method performed much better than under-sampling and hybrid sampling techniques. Among the over-sampling techniques, random sampling has obtained 99% accuracy using XGBoost classifier and 98% accuracy using the decision tree. Besides, it is observed that different resampling methods performed differently with various classifiers.

## 1. Introduction

Parkinson's disease (PD) is the second fastest-expanding neurological chronic condition worldwide. It is a disorder of the brain that leads to stiffness, difficulty in walking, shaking, and loss of balance as mentioned in Program (2021). According to the National Institute of Aging, the prevalence rate of PD is 10 million of the population around the globe affecting 50% more men than women (Ou et al., 2021). Every 5 h, 10 patients are diagnosed with PD. In the United Kingdom, 60,000 people yearly are diagnosed with PD cases before the age of 50 (Parkinson's Change attitudes.Find a cure. Join us, 2020). The estimated PD rate is to double by 2030 (Okunoye et al., 2020).

According to the National Institutes of Health (NIH), rest tremor is the most frequent and quickly recognized symptom. It represents rhythmic, unilateral involuntary, and alternating movements in supported and relaxed upper and lower limbs majorly in the hands, chin, legs, lips, and jaw (Shahed and Jankovic, 2007). Additionally, postural tremor happens when an individual tries to balance their position against the floor gravity, for example expanding and stretching arms and legs. The kinetic tremor occurs during voluntary hand movements such as writing or touching any body part. The frequency of kinetic and postural tremors is between 9–12 Hz and 6–9 Hz, respectively (Pierleoni et al., 2014). The severity of tremor often specifies PD severity and progress which further helps to evaluate treatment efficiency. The ground truth and empirical evidence are collected from UPDRS which is a clinical measurement scale assigned by clinicians with numerical values indicating qualitative observations in various sitting and standing postures (Palmer et al., 2010). Presently, UPDRS measures the PD severity score in a range from 0 to 4 which indicates mild, normal, moderate, slight, and severe levels, respectively (Post et al., 2005). However, UPDRS indicates high variability because the assessment of each clinician may vary depending on his skills and expertise such as one scrutineer assigned a low score on 1 day and another scrutineer assigned a high score on another day (Siderowf et al., 2002; Fisher et al., 2016). In this condition, it is tough to interpret two different values. Moreover, the UPDRS assessment process is tedious.

Therefore, many studies have classified tremor severity values using different signal processing and machine learning (ML) techniques (Ramyachitra and Manikandan, 2014; Belić et al., 2019). The subject of the imbalanced distribution of data or the lack of class density in data training is quite challenging because misclassification can result in a wrong assessment (López et al., 2013; Kaur et al., 2019). On the contrary, various techniques have been implemented to resolve the misclassification problem which is further divided into three sections including resampling of data, modification of algorithm, and cost-effective learning approach. Resampling the data involves under and over sampling including a hybrid approach which resolves the issue of misclassification (Sun et al., 2009; Haixiang et al., 2017).

The survey of literature review implies that this is the initial study to implement various resampling techniques governing the implementation of three classifiers XGBoost, KNN, and Decision tree on five severity levels ranging from 0 to 4 which diminishes the misclassification issue.

The paper is arranged as follows: In the section “Literature Review,” a survey study of related work is conferred. Section “Methodology” discusses the proposed techniques including data collection, signal filtering, and its analysis, and extraction of various important features using different resampling techniques and various ML classifiers. It is followed by “Evaluation of Results and Discussion.” In the end, the “Conclusion” sums up the overall paper.

## 2. Related study

In the past few decades, there has been a rapid rise of interest in the area of early diagnosis of PD and quantification of PD symptoms. For instance, the Bazgir et al. (2015) estimated tremor severity for 52 PD cases using a smartphone feature of a triaxial accelerometer. Various features were extracted including median frequency, dispersion frequency, fundamental tremor frequency, and power spectral density. Additionally, significant results were obtained using an artificial neural network classifier and achieved 91% accuracy. Specifically, no other performance metrics were discussed, the fact that are important for medical classification. Subsequently, Bazgir et al. (2018) improved the performance accuracy using the Sequential Forward Selection approach for feature extraction and achieved 100% using Naive Bayesian classifier.

In Niazmand et al. (2011), the authors experimented on 10 PD cases and two normal control subjects to evaluate tremor severity using integrated pullover triaxial accelerometers. The tremor assessment and peak detection technique were used to calculate the movement frequency. The accelerometers and UPDRS scores were calculated presenting 71 and 89% sensitivity in detecting the correlation of rest tremor and posture tremor, respectively. On the contrary, the pullover i.e., smart clothes that fit completely on the patients attained good results but indicated a fence (obstacle) in routine and continuous PD assessment. Moreover, fit pullover usage can increase the posture tremor muscle tension which shifts the position of the accelerometer depending on the executed movements. Thus, this study provides limited information about patients having UPDRS severities which can impact the performance measurements. Rigas et al. (2012) used wearable accelerometer sets on arms to estimate tremor severity on a range from 0 to 3 involving 10 PD cases, 8 PD cases without tremors, and 5 cases of healthy subjects while performing activities of daily living. Furthermore, low and band pass filters of 3–12 Hz cut off were used for processing. Various sets of features were extricated from filtered signals i.e., low and high frequencies energy, dominant frequency, mechanical energy, and spectrum entropy. Hidden Markov Models were performed for severity classification with Leave-One-Out Cross-Validation (LOOCV) and achieved overall 87% accuracy. As a result, for tremor 0, the achieved specificity and sensitivity were 94 and 91%, respectively. For tremor 1, the achieved specificity and sensitivity were 82 and 87%, respectively, for tremor 2, there were obtained 79 and 69%, respectively, and tremor 3 got percentages of 83 and 91%, respectively. Concurrently, the study is not generalized and applicable to all the patients because tremor severity 4 is missing from the collected data.

Wagner et al. (2017) gathered triaxial accelerometer data using a smartwatch while performing five motor activities including hand rotation, sitting quietly, drawing, folding towels, and walking from 19 patients with PD. Each of the triaxial accelerometer axes including mean relative energy, extracted relative energy, and the acquired signal was processed using the wavelet features extraction technique. Tremor severity was predicted using a support vector machine classifier with three extracted features into 0, 1, and 2 tremor levels representing tremor in one axis only. Therefore, LOOCV was used for model evaluation which achieved 78.91% accuracy, an average recall of 79%, and average precision of 67%. However, a major problem in this study is the combination of 2, 3, and 4 levels of severity into one single score severity which will be a problem to identify tremor level by neurologists later on as it does not represent the actual severity level.

Furthermore, patients with PD were identified from healthy patients using over sampling techniques on speech signals (Polat, 2019). Researchers combined over sampling technique with RF classifier and achieved 94.89% accuracy but the study has not identified the tremor severity. AlMahadin et al. (2022) improved the classification of Parkinson's disease tremor severity by investigating a set of resampling techniques and signal processing techniques. Various resampling techniques were combined with various classifiers including multi-layer perceptron using random forest and ANN. Thus, the results depict that the oversampling performance was better than other techniques of resampling and achieved 93.81% accuracy. However, the sample is very small and does not reflect the whole population. Also, data were collected from one environment source only. Therefore, if the environment is altered then the outcome will definitely vary. On the other hand, the proposed techniques must be experimented with on many other various datasets to measure performance.

In conclusion, a frequent limitation in most of the aforementioned studies is the imbalanced class distribution and the fact that the researchers did not utilize all tremor levels. Only a few studies used data while the subject is performing different tasks, which were specific, and did not include ADLs (activities of daily living) and performance measurements such as AUC (area under the curve), specificity, sensitivity, and F1 score to evaluate the classification models.

## 3. Methodology

**Figure 1:**
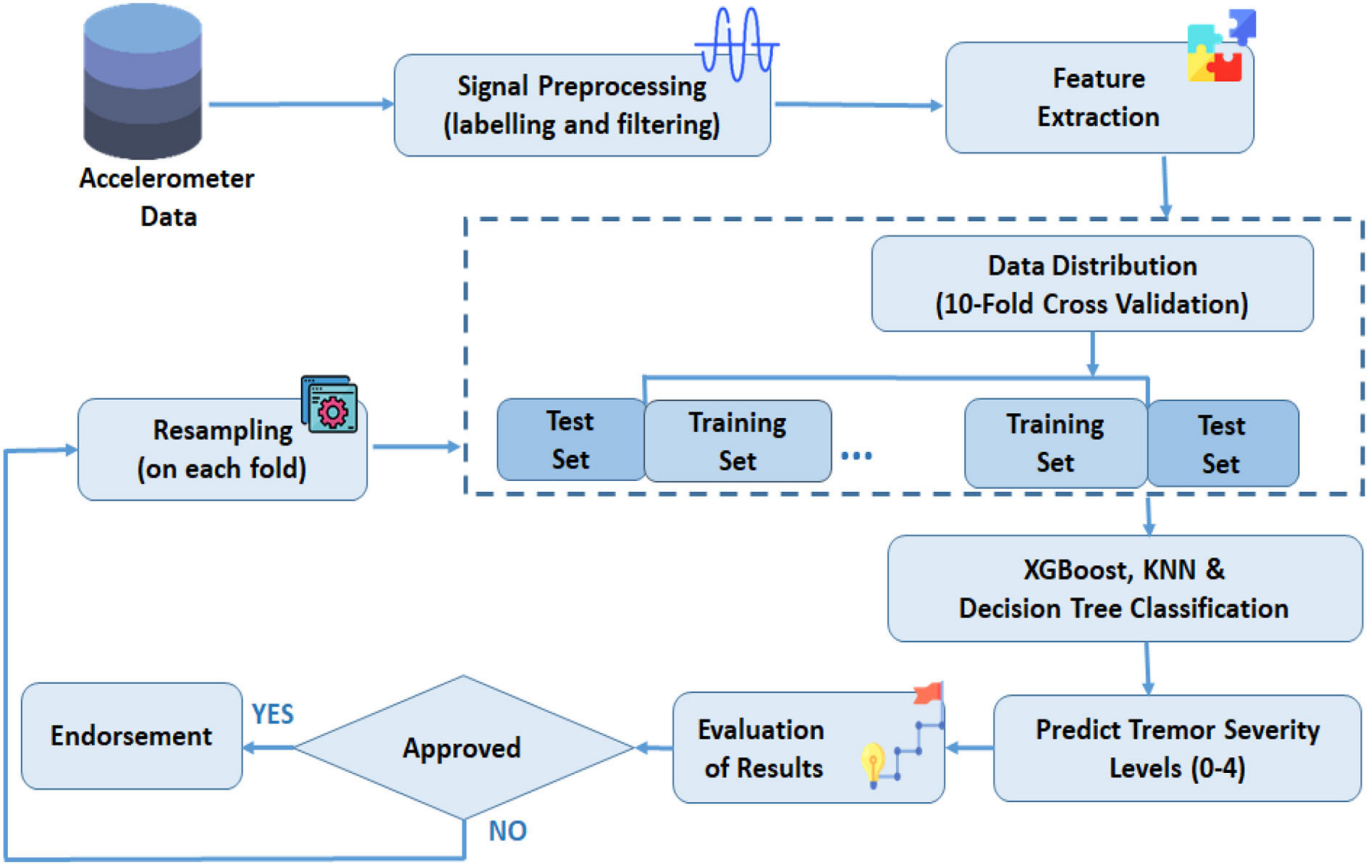
The proposed framework for tremor severity classification.

The proposed strategy to classify the imbalanced RT severity dataset by adapting resampling techniques is depicted in Figure 1. Initially, the raw inertial signals are preprocessed to remove sensor orientation dependency, non-tremor data, and artifacts. In the next step, the time and frequency domain features are extracted from the labeled and prepossessed signals. In the third step, data is segregated into training and test subsets. Additionally, to avoid classifier bias training, the data is resampled. The data distribution is based on 10-fold cross-validation. Finally, tremor severity levels (0–4) are estimated by passing training and test data into a classifier. Furthermore, the results are estimated for adoption in the last step. Each step is explained further in subsequent sections.

### 3.1. Dataset description

The data analyzed in this study is MJFF Levodopa Wearable Sensors Dataset supported by the Michael J. Fox Foundation (https://www.michaeljfox.org/data-sets) (Sage Bionetworks, 2019). The demographic details of the subjects recruited from the Boston study site are given in Table 1. The data is collected from participants in-clinic and home settings using various wearable devices i.e., a Pebble Smartwatch, Shimmer3, GENEActiv accelerometer, and a Samsung Galaxy Mini smartphone accelerometer. These sensors are carried by the patients for the duration of the study i.e., 4 days. In this study, only Shimmer3 accelerometer tremor data collected from subjects in the ON state is analyzed.

**Table 1:** Demographic details of patients with PD.

**Patient ID**	**Gender**	**Age**	**Dominant hand**	**Most effected side**
3BOS	Female	86	Right	Right
4BOS	Female	52	Right	Right
5BOS	Male	74	Right	Right
6BOS	Male	62	Right	Left
7BOS	Male	74	Right	Right
8BOS	Male	64	Right	Right
9BOS	Female	69	Right	Left
10BOS	Male	83	Right	Right
11BOS	Male	61	Right	Right
12BOS	Female	82	Right	Right
13BOS	Male	68	Right	Right
14BOS	Male	65	Right	Right
15BOS	Female	70	Right	Right
16BOS	Male	70	Right	Bilateral
17BOS	Female	60	Left	Bilateral
18BOS	Male	65	Right	Right
19BOS	Male	77	Right	Right

On the first day of data assortment, patients came to the laboratory at their normal recommended routine in ON state (taking medication) and carried out some ADLs, wearing some items for motor assessment in case of movement disorder (Goetz et al., 2008). The rundown of accomplished activities regards standing, strolling straight, strolling while counting, strolling higher up, strolling ground floor, strolling through a narrow entry, finger to nose test, repeated arm development, sit-to-stand, drawing on a paper, composing on paper, assembling nuts and bolts, taking a glass of water, drinking, arranging sheets in an envelope, folding a towel, and sitting. For each instance of each task performed, clinical labels of symptom severity scores (0–4) and/or symptom presence were determined by a medical practitioner. On the second and third days, accelerometer data were gathered while subjects are at home and regulating their usual home activities. Once more, on the fourth day, the same strategy done on the first day was performed, but the patients were in an OFF state (without medication).

### 3.2. Signal preprocessing and features extraction

There are three types of tremors with the following frequency ranges: 3–6 Hz for Resting Tremor (RT), 6–9 Hz for Postural tremor, and 9–12 Hz for Kinetic tremor. Among these, RT presents in 70–90% of patients with PD, and it occurs at a frequency between 4 and 6 Hz. Preprocessing was undertaken to discard non-informative and noisy data. Additionally, the study is based on severity analysis of RT, and eliminates low and high-frequency components from data, holding the RT bands from the signal. As recommended by the previous study, a bandpass Butterworth filter has been used having cut-off frequencies of 3–6 Hz for resting tremor. We employed a 3 s window length for the tremor classifier in view of earlier studies (Patel et al., 2009; Banos et al., 2014). as expressed in Equation (1).


(1) $$\begin{array}{l} {\left\{x_{t}\right\}}_{t=0}^{W_{l}} \end{array}$$

where *x*_*t*_ is the acceleration at a particular time t, and *w*_*l*_ represents the length of the window. The filtered signals are then divided into 3-s windows that can be annotated and applied as inputs. We employed a 3 s window length for the tremor classifier in view of earlier studies (Patel et al., 2009; Banos et al., 2014). In the area of analyzing PD motor symptoms and human action recognition, this window length has shown that it gives a sufficient resolution for separating significant temporal and spectral domain features. As RT lies in between 3 and 6 Hz, a 3 s window length ought to be sufficient for catching important features that distinguish RT. A feature vector comprised of frequency-domain features as well as time-domain features that are computed on each data window. Then, the raw data signal was transformed using the Fast Fourier Transform from the time domain to the frequency domain according to Equation (2) provided below.


(2) $$\begin{array}{l} F\left(y\right)=\overset{W_{l}-1}{\underset{t=0}{\sum }}x_{t}e\frac{-j2\lambda yt}{W_{l}} \end{array}$$

for y = 0....*W*_*l*_-1. F(y) is a complex series that has identical dimensions to the input sequence ${\left(x_{t}\right)}_{t=0}^{W_{l}}$ and $e\frac{-j2\lambda yt}{W_{l}}$ is a primitive Nth root of unity. The extracted features were acquired by processing the acceleration values of the X-, Y-, and Z-axis. The first principal component was calculated from the filtered signals. The first principal component was incorporated as a processed signal for feature extraction to reduce dependence on device orientation. The features were carefully extracted to give detailed and discriminatory information on data characteristics that are highly correlated with tremor severity, such as root mean square (RMS), spectral flatness, entropy, dominant frequency, entropy, autocorrelation, skewness, kurtosis, central tendency, degree of dispersion, and shape of the data. After feature extraction, feature selection is performed. Feature selection has the potential to allow the entire strategy to be executed computationally more productively. Second, it usually achieves an increase in the accuracy or the right rate of the technique. In previous years, several feature selection approaches were proposed. In this study, principal component analysis (PCA) is employed for feature selection. PCA is an efficient method that selects several important features from all the extracted feature components. While assessing the significance of the feature components, the proposed approach considers various eigenvectors. Then it uses a reasonable scheme to perform feature selection.

### 3.3. K-fold cross-validation

In dataset training and testing, the performance of a classifier is evaluated using k-fold cross-validation. Initially, the training set is divided into k-folds of the equal size employing random sampling excluding repetition. Therefore, K times the models are trained, each of them practicing as the training set K as a validation set. The mean average of K individual errors is the prediction error. Moreover, the stability of a model is measured using error variance. The benefit of this technique is that it does not focus more on how the dataset is divided, the model is slighter prone to adapt to the selection bias.

### 3.4. Classifiers

In this study, three classifiers are considered for classification: XGBoost Classifier (Chen and Guestrin, 2016), K-nearest neighbor (KNN) (Peterson, 2009), and Decision Tree (Brijain et al., 2014). The following classifiers were chosen based on the previous study (Parziale et al., 2019; Abdurrahman and Sintawati, 2020; Channa et al., 2021) that attained good accuracy in PD classification and its severity diagnosis with balanced and imbalanced datasets. XGBoost is known as ‘Extreme Gradient Boosting' since it is an ideal blend of hardware and software methods of optimization to yield predominant outcomes incorporating few computing resources in a small time period. XGBoost is a decision-tree-based ensemble Machine Learning (ML) algorithm that utilizes a gradient boosting approach.

However, a Decision Tree is a kind of supervised ML classification algorithm that helps in deciding what the input is in correspondence with a certain output in the training data, where the data is continuously split according to a certain parameter. The tree can be explained by two entities, namely decision nodes and leaves (James et al., 2013). Decision nodes are utilized to build any decision and have various branches, while the leaf nodes represent the outputs of the decisions and do not consist of any branches further. The KNN is a non-parametric supervised learning method that is used for both classification and regression problems. KNN has instance-based learning meaning that it does not learn weights from training data to predict output (as in the model-based algorithms) but uses the entire training instances to predict output for unseen data. The results were estimated using various traditional metrics involving specificity, accuracy, F1-score, precision, and sensitivity (He and Garcia, 2009).

The primary advantage of XGBoost includes various hyper parameters which can be tuned and it has a built-in feature to adjust the missing values. Also, it provides distributed computing, parallelism, and cache optimization.

### 3.5. Adopted resampling methods

The resampling methodology is adopted when there is an unbalanced dataset to improve the accuracy and quantify the uncertainty. Resampling techniques alter the formation of a dataset training for an imbalanced classification quest. This section confers a brief overview of the resampling strategies utilized in this study. Resampling techniques can be divided into three groups: under-sampling, over-sampling, and hybrid (merging over and under-sampling).

#### 3.5.1. Under-sampling techniques

**Figure 2:**
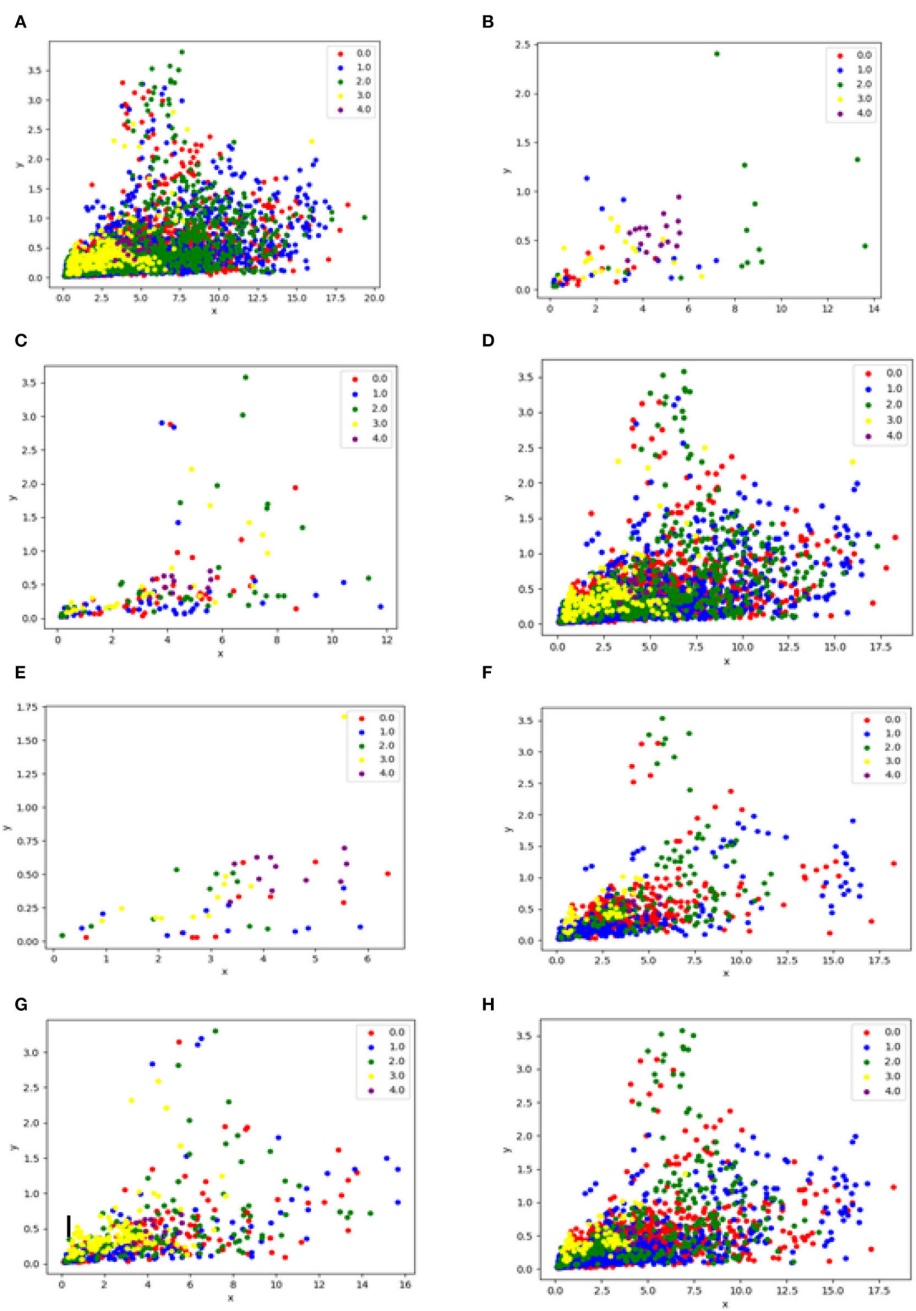
This figure illustrates the difference before and after applying the under-sampling technique, **(A)** is data without resampling, **(B)** is data after applying the Random undersampling method, **(C)** is data after applying the CNN technique, **(D)** is data after applying the Tomek Links method, **(E)** is data after applying NearMiss method, **(F)** is data after applying the ENN resampling method, **(G)** is data after applying the OSS resampling method, **(H)** is the data after applying the NCR resampling method.

In Under-sampling methods, the samples taken from the majority of classes are reduced. In this proposed framework, seven under-sampling techniques are investigated as explained below and Figure 2 depicts the difference between all of them.

##### 3.5.1.1. Random undersampling

Random undersampling (Hoens and Chawla, 2013; Liu and Tsoumakas, 2020) includes randomly selecting examples from the majority class and eliminating them from the training dataset. In this methodology, the greater part of class occurrences is disposed of arbitrarily until a more adjusted dispersion is reached.

##### 3.5.1.2. Condensed nearest neighbor

This technique was proposed by Hart (1968) to decrease the memory prerequisites for the K-Nearest Neighbors (KNN) approach. It looks for a subset of an assortment of samples that results in no loss in model execution, alluded to as a minimal consistent set. This is accomplished by multiple iterations upon the majority of classes and choosing samples of subsets that are accurately classified by the algorithm known as 1-nearest neighbor. Therefore, it considers only the relevant number of samples and excludes the insignificant samples from the majority of classes.

##### 3.5.1.3. Tomek links

As the CNN technique initially selects random samples, this produces retention of irrelevant samples and occasional retention of internal instead of boundary samples. Hence, the modification of the CNN procedure proposed by Two Modifications of CNN (1976) and called Tomek Links finds the pairs of various examples, one taken from each of the classes that have the Euclidean distance of smallest value to each other, in features space. Then it removes the samples belonging to the majority of classes or vice versa. In this study, the majority of Tomek Links classes are discarded only to retain the minority classes and to increase the distances between the classes by discarding the majority classes near the decision boundary.

##### 3.5.1.4. NearMiss

Near Miss refers to an assortment of undersampling strategies that selects the models in light of the distance of the larger part class to the minority class models. This approach was proposed by Mani and Zhang (2003). There are three sorts of methods, named NearMiss-1, NearMiss-2, and NearMiss-3. NearMiss-1 picks the larger part class models with the average distance which is the smallest to the three nearest minority of class models, NearMiss-2 chooses the larger part class tests with the average distance which is smallest to the three farthest minority of samples, NearMiss-3 chooses greater part class tests with the distance which is smaller to each minority class sample. Therefore, in this research NearMiss-1 is employed.

##### 3.5.1.5. Edited nearest neighbor

Edited Nearest Neighbor (ENN) (Wilson, 1972) is a technique for tracking uncertain and noisy samples in a dataset. This technique uses the *k* = 3 closest neighbors to find those models in a dataset that are misclassified, and those are then eliminated before a *k* = 1 order rule is implemented. In the undersampling system, the standard is employed for each model in the majority of the class, permitting those models which are misclassified as existing to the minority class to be taken out and accurately classified to remain.

##### 3.5.1.6. One sided selection

One-Sided Selection or OSS (Kubat and Matwin, 1997) joins Tomek Links and the Condensed Nearest Neighbor (CNN) rule. This aims to eliminate the models from the larger part class that are distant from the decision border. In particular, the Tomek Links technique provides an uncertain number of points on the boundary of a class and these points are recognized and taken out in the larger part class. Therefore, the CNN step takes place in one stage and consists initially of adding the entire minority class examples to the store and a few majority class examples (e.g., 1) later on, classifying the left over majority class examples with KNN (k = 1) and summing those which are misclassified to store.

##### 3.5.1.7. Neighborhood cleaning rule

This methodology of resampling and classification i.e., Neighborhood Cleaning Rule, or NCR (Laurikkala, 2001) focuses barely on improving the equilibrium of a class dispersion and erring in the quality (unambiguity) of the models that are held in the greater part class. The strategy includes first choosing all models from the minority of class. Later, the entire uncertain examples in the majority class are recognized by applying the ENN rule and are eliminated. At last, a version of one-step CNN is involved where those left over examples in the other majority class that appears to be misclassified as opposed to the store are discarded and the number of examples in the majority class is larger than half of the size of the minority class.

#### 3.5.2. Over-sampling techniques

**Figure 3:**
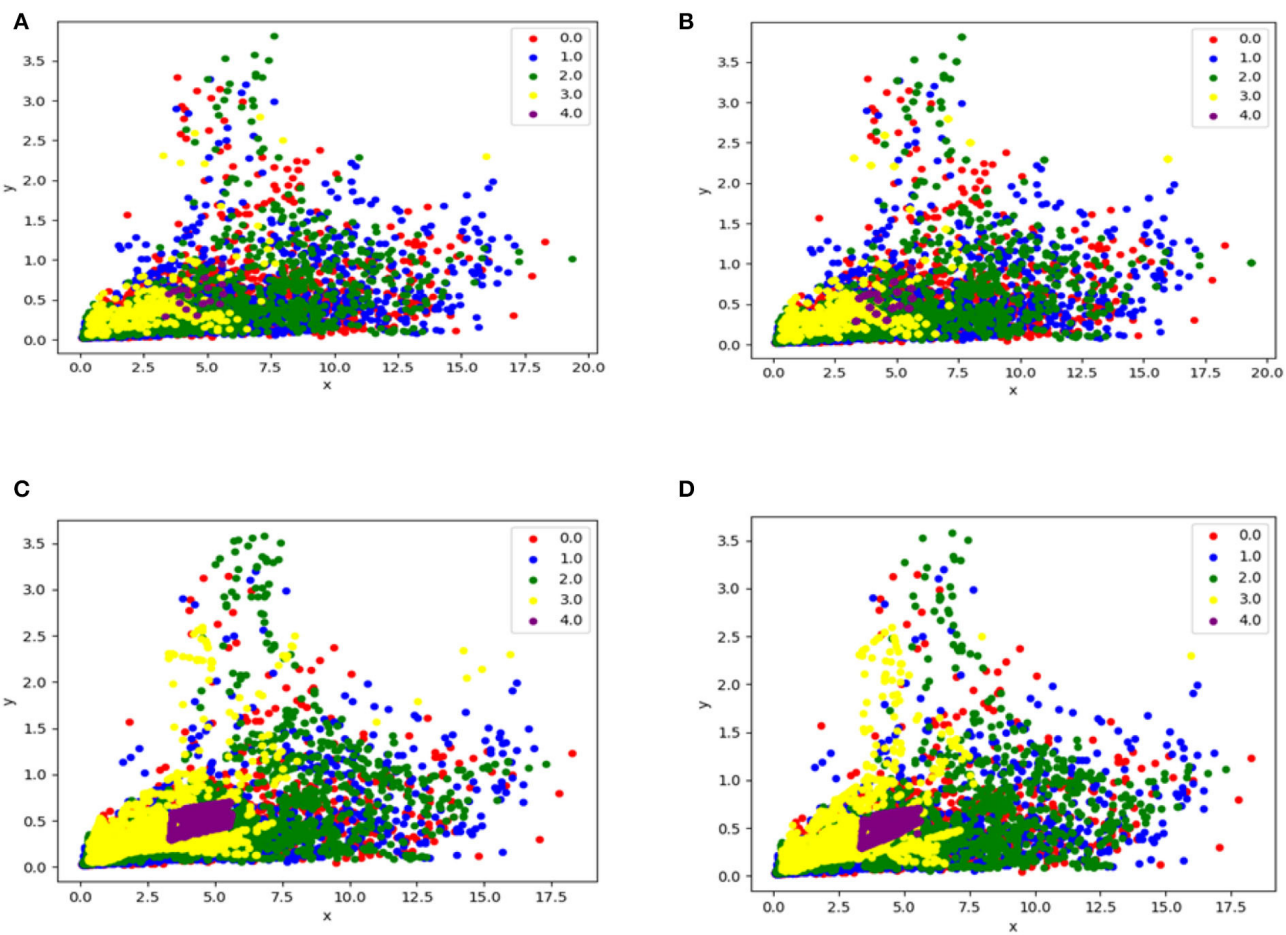
This figure illustrates the difference between before and after cases using over-sampling techniques, **(A)** is data without any resampling method, **(B)** is data after applying Random-oversampling resampling method, **(C)** is data after applying SMOTE resampling, **(D)** is data after applying Borderline SMOTE method.

In Over-sampling techniques, the samples of the minority of classes are increased. In this research study, three over-sampling techniques are explored as discussed below. Figure 3 depicts the difference between all of them. Since oversampling proved to be right approach for our problem, hence, to avoid overfitting or performing resampling in the wrong way, the oversampling is done during cross-validation, i.e., for each fold, oversampling is performed before training, and this process is repeated for each fold.

##### 3.5.2.1. Random oversampling

Random oversampling (Hoens and Chawla, 2013) involves randomly selecting examples from the minority class, with replacement, and adding them to the training dataset.

##### 3.5.2.2. Synthetic minority over-sampling technique

The SMOTE approach (Chawla et al., 2002) synthetically generates several samples in the minority class rather than replacing the original number of samples, which returns to an over-fitting problem. Moreover, SMOTE generates samples that are based on similarities in the feature space with the line segments combining the minority instances and feature space containing ‘k' minority class nearest neighbors.

##### 3.5.2.3. Borderline SMOTE

The SMOTE borderline (Han et al., 2005) recognizes decision boundary (borderline) minority samples and then SMOTE algorithm is implemented to create synthetic samples along with decision the boundary of majority and minority classes.

#### 3.5.3. Combine resampling techniques or hybrid resampling methods

There are various combinations of under and over sampling techniques which have proven to be more effective and together may be expressed as the resampling method.

**Figure 4:**
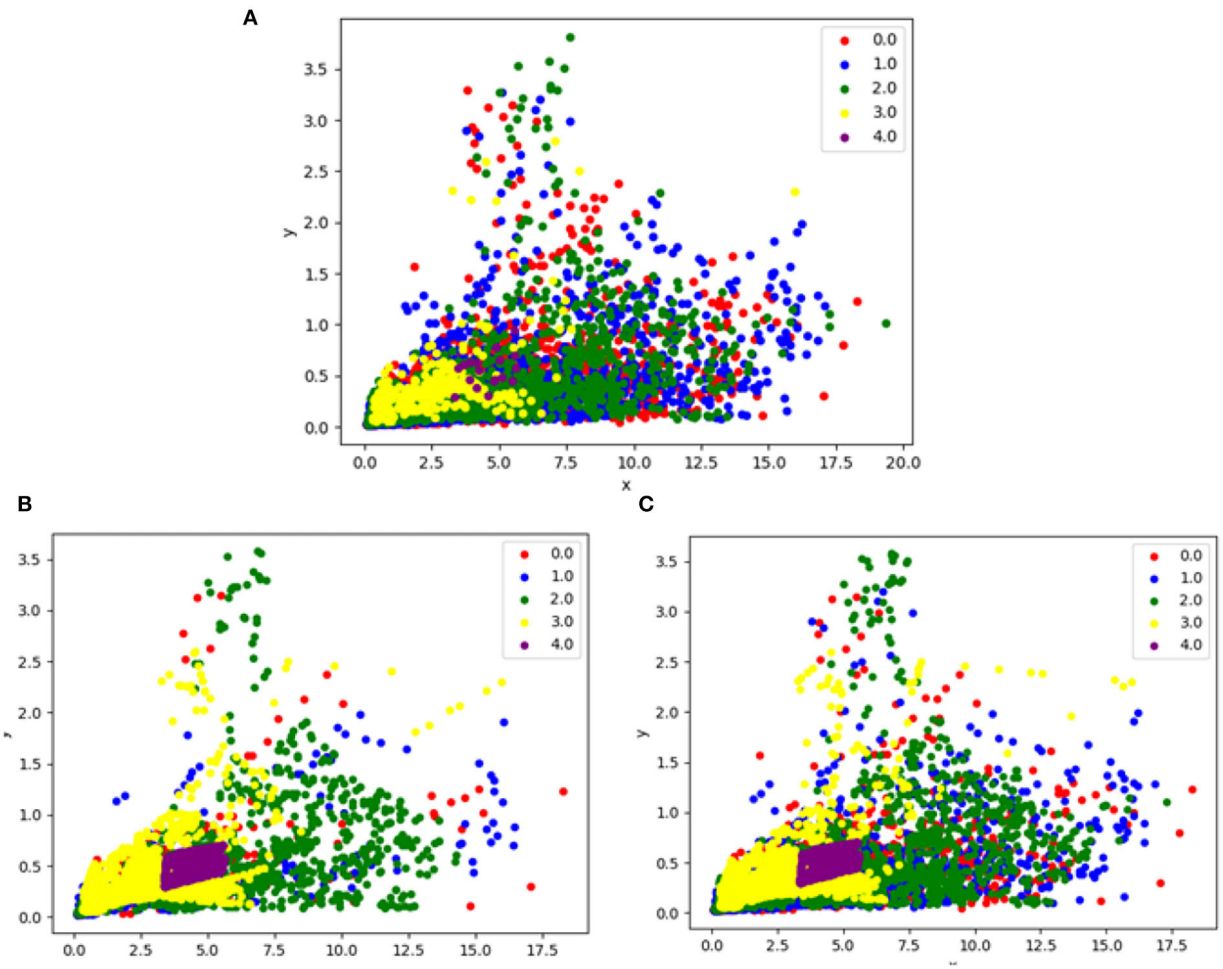
This figure illustrates the difference between before and after hybrid resampling, **(A)** is data without resampling, **(B)** is data after applying the SMOTETOMEK resampling method, and **(C)** is data after applying the SMOTEENN resampling method.

Therefore, two examples are given as mixtures of SMOTE with Edited Nearest Neighbors undersampling and SMOTE with Tomek Links undersampling. Figure 4 depicts the difference between them.

##### 3.5.3.1. SMOTE with tomek link

Synthetic Minority Over-sampling Technique is known as an oversampling technique that incorporates advanced plausible examples in the minority class. Tomek Links denotes a technique for recognizing pairs of nearest neighbors in a dataset that has various classes. Discarding both or one of the examples in these pairs (such as the examples in the majority class) has the effect of forming the decision boundary in the dataset, training uncertain and slighter the noise. The combination (Batista et al., 2003) provides a reduction in false negatives at the cost of an increase in false positives for a binary classification task.

##### 3.5.3.2. SMOTE combined with ENN

Synthetic Minority Over-sampling Technique may be considered the most prominent oversampling method and can be merged with various undersampling methods. ENN is more intrusive at downsampling the majority class than the Tomek Links method, giving more in-depth cleaning. The method was implemented by discarding different examples from both minority and majority classes. Hence, this combination (Batista et al., 2004) yielded amazing results in practice.

## 4. Results and discussions

First, the raw signal was labeled to individual procession events through a Python script with the 3-s window preceding motion onset. Table 2 represents the class (severity levels) distribution of 7,740 instances (windows) divided from the collected data using the Shimmer3 device. Therefore, it is clearly observed how the distribution of data is skewed in the case of less severe tremors. This bias can result in significant changes in classification output. In these circumstances, the classifier is extra sensitive in identifying the majority of classes but it becomes less sensitive to recognize the minority of classes if they are excluded.

**Table 2:** Imbalanced data distribution.

**Class (RT severity)**	**Instances**	**After under-sampling**	**After oversampling**
0	3,170	18	2,205
1	2,894	18	2,205
2	1,391	18	2,205
3	291	18	2,205
4	4	18	2,205

Thus, different resampling techniques are utilized to eliminate the imbalanced class distribution effect. Table 2 depicts the number of imbalanced instances and the number of balanced instances after resampling techniques. The results of different resampling techniques are described below:

### 4.1. Under-sampling evaluation

Table 3 explains the performance of three classifiers i.e., XGBoost, decision tree, and KNN on the PD RT severity dataset. Seven resampling techniques: Random, ENN, NearMiss, CNN, Tomeklinks, OSS, and NER are utilized. From the results, it is clearly observed that all classifiers show the worst performance using undersampling methods as compared to the case without following any resampling method. However, some under-sampling techniques improved the performance of the classifiers. All three classifiers achieved the best performance with only the TomekLink under-sampling technique. The best results have been got with the XGBoost classifier with TomekLink obtaining 68% accuracy, while the random resampling method along with NearMiss and CNN is significantly worst.

**Table 3:** Performance metrics with/without Under-sampling techniques for resting tremor severity classification with XGBoost, decision tree, and KNN.

			**Under-sampling technique**						
**Classifier**	**Matrics**	**W/o sampling**	**Random**	**ENN**	**NearMiss**	**CNN**	**Tomeklinks**	**OSS**	**NER**
XGBoost	Accuracy (%)	70	30	59	28	38	68	53	66
	Precision Score 0	0.76	0.45	0.63	0.48	0.50	0.72	0.65	0.69
	Precision Score 1	0.67	0.45	0.53	0.41	0.45	0.65	0.54	0.63
	Precision Score 2	0.63	0.22	0.70	0.16	0.28	0.66	0.40	0.70
	Precision Score 3	0.59	0.11	0.50	0.09	0.10	0.63	0.26	0.64
	Precision Score 4	1.0	0.09	0.71	0.02	0.12	1.0	0.27	1.0
	F1-score 0	0.79	0.31	0.65	0.31	0.43	0.77	0.62	0.75
	F1-score 1	0.68	0.35	0.60	0.40	0.41	0.67	0.53	0.65
	F1-score 2	0.52	0.27	0.41	0.14	0.33	0.51	0.39	0.46
	F1-score 3	0.50	0.20	0.33	0.15	0.14	0.52	0.38	0.41
	F1-score 4	0.25	0.17	0.71	0.03	0.20	0.44	0.33	0.73
Decision Tree	Accuracy (%)	55	29	50	30	28	54	40	54
	Precision Score 0	0.65	0.44	0.56	0.43	0.44	0.62	0.53	0.57
	Precision Score 1	0.56	0.44	0.47	0.55	0.40	0.55	0.49	0.54
	Precision Score 2	0.37	0.26	0.42	0.18	0.25	0.41	0.28	0.45
	Precision Score 3	0.39	0.07	0.38	0.07	0.08	0.20	0.13	0.35
	Precision Score 4	0.50	0.06	0.07	0.02	0.02	0.0	0.09	0.67
	F1-score 0	0.65	0.34	0.56	0.48	0.28	0.63	0.48	0.61
	F1-score 1	0.55	0.35	0.52	0.19	0.37	0.55	0.43	0.55
	F1-score 2	0.37	0.27	0.32	0.12	0.29	0.39	0.31	0.36
	F1-score 3	0.39	0.12	0.28	0.12	0.13	0.22	0.21	0.28
	F1-score 4	0.36	0.11	0.10	0.03	0.03	0.0	0.14	0.49
KNN	Accuracy (%)	51	39	51	28	41	51	46	51
	Precision Score 0	0.52	0.47	0.52	0.39	0.45	0.52	0.52	0.51
	Precision Score 1	0.51	0.40	0.50	0.44	0.40	0.51	0.48	0.51
	Precision Score 2	0.50	0.30	0.52	0.16	0.21	0.51	0.37	0.52
	Precision Score 3	0.45	0.15	0.48	0.12	0.16	0.44	0.20	0.46
	Precision Score 4	1.0	0.24	0.43	0.01	1.0	1.0	1.0	0.50
	F1-score 0	0.61	0.47	0.60	0.32	0.52	0.61	0.55	0.62
	F1-score 1	0.47	0.36	0.46	0.35	0.37	0.46	0.44	0.44
	F1-score 2	0.33	0.29	0.32	0.29	0.15	0.32	0.31	0.31
	F1-score 3	0.26	0.22	0.25	0.16	0.07	0.25	0.28	0.21
	F1-score 4	0.25	0.39	0.43	0.02	0.44	0.25	0.25	0.22

### 4.2. Over-sampling evaluation

Table 4 explains the performance of three classifiers i.e., XGBoost, decision tree, and KNN on the PD RT severity dataset. Three resampling techniques: Random, ENN, SMOTE, and Borderline SMOTE are employed. In general, all the implemented techniques of over-sampling improved the performance of the classifiers significantly. Also, it can be noted that the performance of the XGBoost classifier is better than the decision classifier using over-sampling technique whereas the decision tree classifier achieved better results than KNN without using the over-sampling. On the contrary, the best results were achieved using the resampling method undertaken by random sampling. Using the random sampling technique decision tree gives maximum accuracy i.e., 100%, XGBoost gives 98% and KNN gives 87%. However, SMOTE and Borderline SMOTE performance results are almost the same. These results highlight that the over sampling techniques performance is much better than under sampling techniques.

**Table 4:** Performance metrics with/without Over-sampling techniques for resting tremor severity classification with XGBoost, decision tree, and KNN.

			**Over-sampling technique**		
**Classifier**	**Matrics**	**Without sampling**	**Random**	**SMOTE**	**Borderline SMOTE**
XGBoost	Accuracy (%)	70	99	69	69
	Precision Score 0	0.76	0.99	0.76	0.75
	Precision Score 1	0.67	0.99	0.66	0.67
	Precision Score 2	0.63	1.0	0.60	0.59
	Precision Score 3	0.59	1.0	0.52	0.51
	Precision Score 4	1.00	1.0	0.60	1.00
	F1-Score 0	0.79	0.99	0.78	0.78
	F1-Score 1	0.68	0.99	0.67	0.66
	F1-Score 2	0.52	0.99	0.55	0.53
	F1-Score 3	0.50	1.00	0.57	0.57
	F1-Score 4	0.25	1.00	0.50	0.44
Decision Tree	Accuracy (%)	55	98	51	55
	Precision Score 0	0.65	0.98	0.62	0.67
	Precision Score 1	0.56	0.99	0.52	0.55
	Precision Score 2	0.37	0.99	0.36	0.36
	Precision Score 3	0.39	1.0	0.22	0.33
	Precision Score 4	0.50	1.0	0.75	33
	F1-Score 0	0.65	0.98	0.61	0.64
	F1-Score 1	0.55	0.98	0.50	0.54
	F1-Score 2	0.37	0.99	0.37	0.40
	F1-Score 3	0.39	1.0	0.29	0.38
	F1-Score 4	0.36	1.0	0.55	0.20
KNN	Accuracy (%)	51	87	51	51
	Precision Score 0	0.52	0.79	0.55	0.54
	Precision Score 1	0.51	0.96	0.52	0.52
	Precision Score 2	0.50	1.0	0.43	0.42
	Precision Score 3	0.45	1.0	0.37	0.40
	Precision Score 4	1.00	1.0	0.50	0.50
	F1-Score 0	0.61	0.88	0.60	0.60
	F1-Score 1	0.47	0.83	0.46	0.40
	F1-Score 2	0.33	0.92	0.38	0.38
	F1-Score 3	0.26	1.0	0.44	0.45
	F1-Score 4	0.25	1.0	0.59	0.53

### 4.3. Hybrid sampling evaluation

Table 5 exhibits the performance of three classifiers i.e., XGBoost, decision tree, and KNN on the PD dataset of resting tremor severity. Two hybrid resampling techniques: SMOTEENN and SMOTETomek are employed. In contrast to over-sampling techniques, both hybrid techniques and under-sampling techniques did not improve classifiers' performance significantly. But the SMOTETOMEK performance with both classifiers worked better than SMOTEENN. SOMTEENN got the best results with the XGBoost classifier with 68% overall accuracy which is even lesser than the accuracy of XGBoost without following any resampling method.

**Table 5:** Performance metrics with/without Hybrid resampling techniques for resting tremor severity classification with XGBoost, decision tree, and KNN.

			**Hybrid sampling technique**	
**Classifier**	**Matrics**	**Without sampling**	**SMOTETomek**	**SMOTEENN**
XGBoost	Accuracy (%)	70	68	49
	Precision Score 0	0.76	0.78	0.75
	Precision Score 1	0.67	0.64	0.56
	Precision Score 2	0.63	0.59	0.32
	Precision Score 3	0.59	0.51	0.25
	Precision Score 4	1.0	0.75	0.75
	F1-Score 0	0.79	0.77	0.55
	F1-Score 1	0.68	0.67	0.51
	F1-Score 2	0.52	0.53	0.43
	F1-Score 3	0.50	0.56	0.36
	F1-Score 4	0.25	0.55	0.55
Decision Tree	Accuracy (%)	55	52	39
	Precision Score 0	0.65	0.65	0.65
	Precision Score 1	0.56	0.52	0.49
	Precision Score 2	0.37	0.34	0.26
	Precision Score 3	0.39	0.25	0.17
	Precision Score 4	0.50	1.00	1.00
	F1-Score 0	0.65	0.61	0.44
	F1-Score 1	0.55	0.53	0.42
	F1-Score 2	0.37	0.36	0.35
	F1-Score 3	0.39	0.33	0.27
	F1-Score 4	0.36	0.60	0.73
KNN	Accuracy (%)	51	51	44
	Precision Score 0	0.52	0.56	0.63
	Precision Score 1	0.51	0.52	0.53
	Precision Score 2	0.50	0.40	0.30
	Precision Score 3	0.45	0.36	0.19
	Precision Score 4	1.0	0.45	0.33
	F1-Score 0	0.61	0.59	0.50
	F1-Score 1	0.47	0.49	0.43
	F1-Score 2	0.33	0.36	0.39
	F1-Score 3	0.26	0.42	0.30
	F1-Score 4	0.25	0.56	0.48

## 5. Performance metrics

**Figure 5:**
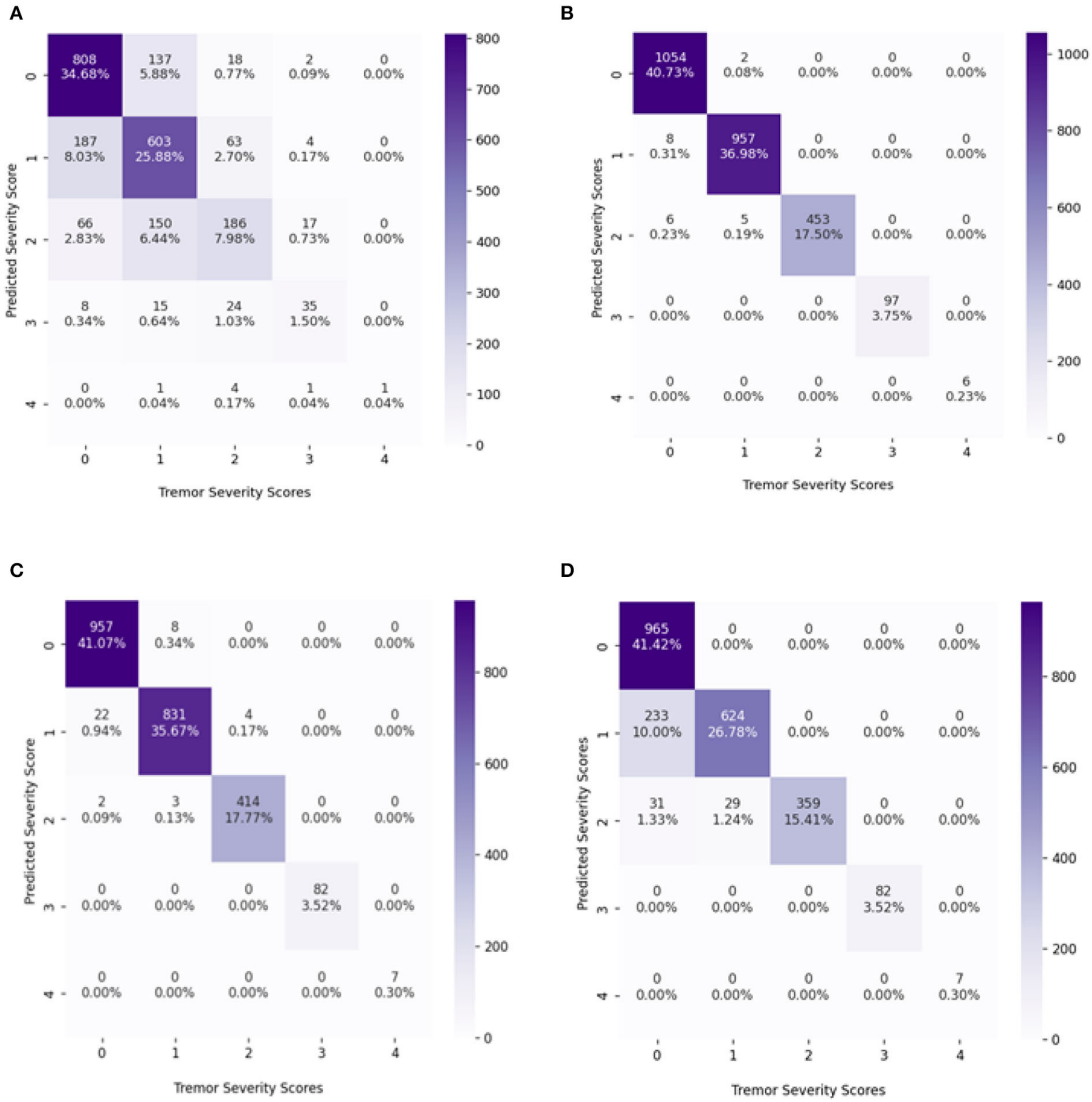
The confusion matrix (CM) of the classifiers with maximum accuracy, **(A)** CM of the XGBoost classifier without resampling, **(B)** CM of the XGBoost classifier with a random over-sampling method., **(C)** CM of Decision tree classifier with a random over-sampling method., **(D)** CM of KNN classifier with a random over-sampling method.

The confusion matrix of the best results obtained so far with and without resampling techniques is shown in Figure 5. Without the resampling method, the XGBoost classifier performed better than KNN and the decision tree. The accuracy obtained using XGBoost without resampling is 70% with KNN 51% and with decision tree 55%. However, with the combination of the resampling techniques, XGBoost performed well getting 99% accuracy, decision tree with 98%, and KNN with 87%. This clearly shows that the classifiers performed well with balanced data in each class as compared to the imbalanced number of samples in classes.

From the confusion matrix, it is observed the accuracies achieved using XGBoost and decision tree using random oversampling: 99 and 98%. There is a slight difference between both classifiers' results. The measurements for assessing the performance of classifiers are precision, accuracy, sensitivity, and specificity. But in many applications specially in the case of analyzing imbalanced data, these metrics are insufficient, since they are related to the data distribution in each class. For the majority classes, sample classifier prediction is based on high accuracy, but for minority classes it is low. Sensitivity and precision do not consider true negatives (TN) in the area of medical diagnosis field where misclassified TN can lead to unnecessary treatment. Similarly, precision can be seen as a measure of quality. Higher precision and sensitivity means that an algorithm returns more relevant results that an irrelevant one. Hence, to balance this fact, F1-score and Gmean (geometric mean) are the ultimate choices to minimize the effect of imbalanced distribution. However, Gmean and F1-score do not take into account the TN and the classes' contribution to overall performance. Therefore, advanced metric i.e., IBA (index of balanced accuracy) is calculated. IBA evaluates the contribution of each class as the overall performance so that high IBA is obtained when the accuracy of all classes is high and balanced (Garćıa et al., 2009). The IBA evaluates the relationship between TPR and TNR, representing the class distribution. IBA can take any value between 0 and 1, and the best performance achieved is got when TPR = TNR = 1 with α = 1. Hence, in this study, advanced metrics are calculated i.e., accuracy, precision, sensitivity, specificity, F1-score, Gmean, and IBA as expressed in Equations below, for the classifiers which show the best performance.


(3) $$\begin{array}{lll} Accuracy & = & \frac{TP+TN}{TP+TN+FP+FN} \end{array}$$

(4) $$\begin{array}{lll} Precision & = & \frac{TP}{TP+FP} \end{array}$$

(5) $$\begin{array}{lll} Sensitivity & = & TPR=\frac{TP}{TP+FN} \end{array}$$

(6) $$\begin{array}{lll} Specificity & = & TNR=\frac{TN}{TN+FP} \end{array}$$

(7) $$\begin{array}{lll} F1 & = & \frac{2\times Precision\times Sensitivity}{Precision+Sensitivity} \end{array}$$

(8) $$\begin{array}{lll} Gmean & = & \sqrt{Sensitivity\times Specificity} \end{array}$$

$$\begin{array}{lll} IBA_{\alpha } & = & 1+\alpha \times \left(TPR-TNR\right) \end{array}$$

(9) $$\begin{array}{ll} \times & GMean^{2},where\;0\;\leq \alpha \leq 1 \end{array}$$

Table 6 explains the performance metrics of the XGBoost classifier with imbalanced data classification. Only class 4 of the data points are predicted well. Some metrics are declined and some metrics are improved; there is no synchronization between the parameters. However, Tables 7–9, regarding the classifiers with a combination of random oversampling techniques, it is clearly shown the prediction of all classes without any bias toward the majority classes. The most striking part about the results is the important metrics i.e., *IBA*_α_ and Gmean are also improved in parallel with the accuracy. The *IBA*_α_ and Gmean of the XGBoost classifier with random over-sampling of all classes are significantly better than the rest of the classifiers and their performance.

**Table 6:** Performance metrics of XGBoost classifier with imbalance data classification.

	**Accuracy**	**Precision**	**Sensitivity**	**Specificity**	**F1-Score**	**Gmean**	**IBA_α_**
Class 0	0.82	0.75	0.83	0.808	0.79	0.81	1.0
Class 1	0.76	0.66	0.70	0.79	0.68	0.74	1.0
Class 2	0.85	0.63	0.44	0.94	0.52	0.64	0.80
Class 3	0.96	0.59	0.42	0.98	0.49	0.64	0.78
Class 4	0.99	1	0.14	1	0.25	0.37	0.89

**Table 7:** Performance metrics of XGBoost classifier with random over-sampling technique.

	**Accuracy**	**Precision**	**Sensitivity**	**Specificity**	**F1-Score**	**Gmean**	**IBA_α_**
Class 0	0.99	0.98	0.99	0.99	0.98	0.99	1.0
Class 1	0.98	0.99	0.99	0.95	0.99	0.96	1.0
Class 2	0.99	1	0.97	1	0.98	0.98	0.98
Class 3	1	1	1	1	1	1	1.0
Class 4	1	1	1	1	1	1	1.0

**Table 8:** Performance metrics of decision tree classifier with random over-sampling technique.

	**Accuracy**	**Precision**	**Sensitivity**	**Specificity**	**F1-Score**	**Gmean**	**IBA_α_**
Class 0	0.98	0.97	0.99	0.98	0.97	0.98	0.99
Class 1	0.98	0.98	0.96	0.99	0.96	0.97	0.97
Class 2	0.99	0.99	0.98	0.99	0.98	0.98	0.99
Class 3	1	1	1	1	1	1	1.0
Class 4	1	1	1	1	1	1	1.0

**Table 9:** Performance metrics of KNN classifier with random over-sampling technique.

	**Accuracy**	**Precision**	**Sensitivity**	**Specificity**	**F1-Score**	**Gmean**	**IBA_α_**
Class 0	0.88	0.78	1	0.80	0.87	0.89	1.1
Class 1	0.88	0.95	0.72	0.98	0.81	0.84	0.82
Class 2	0.97	1	0.85	1	0.91	0.92	0.88
Class 3	1	1	1	1	1	1	1.0
Class 4	1	1	1	1	1	1	1.0

## 6. Conclusion and future directions

In this study, PD resting tremor severity is estimated using different ML classifiers in combination with signal processing and resampling techniques. From the results, it is observed that oversampling techniques performed better than under-sampling and hybrid resampling approaches. Among classifiers, the XGBoost classifier stands better than KNN and decision tree classifiers. However, it is also observed that resampling techniques do not perform similarly with different classifiers. Some important metrics such as accuracy and precision are very low and declined dramatically with some under-sampling techniques, despite that other metrics are improved. Above all, our study investigated individual classes' classification instead of overall performance. This study has some limitations that need to be considered as the sample size can be better chosen. The proposed approach needs more validation on different datasets on PwPD (patients with PD). In the future, large sample size data can be considered. The data analyzed in this study was collected from the most affected limb of PwPD; if the data from both upper-limbs of PwPD is gathered, the results may vary.

## Data availability statement

The datasets presented in this study can be found in online repositories. The names of the repository/repositories and accession number(s) can be found below: https://www.michaeljfox.org/data-sets.

## Ethics statement

Ethical review and approval were not required for the study on human participants in accordance with the local legislation and institutional requirements. Written informed consent was obtained from the individual(s) for the publication of any potentially identifiable images or data included in this article.

## Author contributions

All authors listed have made a substantial, direct, and intellectual contribution to the work and approved it for publication.

## Funding

This research was funded by the European Union's Horizon 2020 Research and Innovation program under the Marie Skodowska Curie grant agreement No. 813278 (A-WEAR: A network for dynamic wearable applications with privacy constraints, http://www.a-wear.eu/), and a grant from the Romanian National Authority for Scientific Research and Innovation, UEFISCDI project PN-III-P3-3.6-H2020-2020-0124).

## Conflict of interest

The authors declare that the research was conducted in the absence of any commercial or financial relationships that could be construed as a potential conflict of interest.

## Publisher's note

All claims expressed in this article are solely those of the authors and do not necessarily represent those of their affiliated organizations, or those of the publisher, the editors and the reviewers. Any product that may be evaluated in this article, or claim that may be made by its manufacturer, is not guaranteed or endorsed by the publisher.
